# A Hint for British Artists

**Published:** 1890-10-04

**Authors:** 


### A Hint for British Artists.

The town of Amsterdam has long needed a new and modern hospital for the accommodation of the sick. Plans have been prepared and approved for such a building, but £12,000 is still needed to complete the sum required to defray the cost of erection. In this emergency a committee has been formed, of which one of the leading citizens, Mr. R. Kyzer, is the honorary secretary, to organize a lottery, and so secure the necessary funds. The Dutch artists have thrown themselves into the movement with commendable zeal, and have offered to give the committee a selection of their best works at half the price they would procure for them if sold in the ordinary way. The whole of the works to be distributed through the lottery will be exhibited in Amsterdam, water-colour drawings and pieces of sculpture being included, as well as oil paintings. The lottery is divided into 150 series, each containing 100 lottery tickets of 10 florins each. An Englishman, well acquainted with Holland, who has been struck with the urgent necessity for a new hospital for Amsterdam, has offered to take up one series providing the remaining 149 are subscribed for on or before December 31st, 1890. Lotteries being illegal in England, it is impossible for hospital secretaries to take this hint, though we are certain that British artists would willingly do anything in their power to support British hospitals. Might we suggest that some of them, at any rate, would help to alleviate the tedium and monotony of hospital wards if they were to send from time to time a painting or water-colour or piece of sculpture to some of our hospitals? Not only would this practice be much appreciated by the authorities, but it could not fail to have other advantages to the artists, and also to increase the love of artistic work.

We wish our Dutch friends a complete success in their laudable efforts.

